# Non-Disclosure of abuse in children and young adults with disabilities: Reasons and mitigation strategies Northwest Region of Cameroon

**DOI:** 10.4102/ajod.v11i0.1025

**Published:** 2022-12-14

**Authors:** Glory T. Tsangue, Jacque Chirac Awa, Josephine Nsono, Charlotte W. Ayima, Pius M. Tih

**Affiliations:** 1 Empowerment and Disability Inclusive Development (EDID) Program, Cameroon Baptist Convention Health Services, Yaounde, Cameroon; 2 Services for Persons with Disability, Cameroon Baptist Convention Health Services, Bamenda, Cameroon; 3 Gender and Child Protection Services, Cameroon Baptist Convention Health Services, Bamenda. Cameroon; 4 Department of Research/Evaluation, Cameroon Baptist Convention Health Services, Mutengene-Buea, Cameroon; 5 Cameroon Baptist Convention Health Services, Bamenda, Cameroon

**Keywords:** children with disabilities, nondisclosure, reasons, mitigation, Northwest Region, Cameroon

## Abstract

**Background:**

Child abuse is a serious public health issue in low- and middle-income countries, and children with disabilities are at greater risk of abuse. Despite this heightened risk, the abuse of children with disabilities often goes undetected and under-reported, leading to the continuity of such abuse by their abusers.

**Objectives:**

This study was aimed at identifying the reasons for non-disclosure of abuse and possible mitigating strategies to curb this dilemma in children and young adults with disabilities (CWD).

**Methods:**

A population-based record-linkage qualitative study was conducted among CWD (both at home and in institutions) in the Northwest Region of Cameroon. Twelve key informant interviews and eight focus group discussions (FGDs) were conducted among key staff from child protection offices for child abuse, parents and teachers in schools. Fifty in-depth interviews were also conducted among children with disabilities. Reasons for nondisclosure and proposed mitigating approaches from audio tapes were transcribed verbatim, thematic analysis performed and findings reported.

**Results:**

A lack of knowledge on where to disclose, fear of stigma, long and expensive procedures, a lack of confidence in the justice system, threats from abusers, protection of family unity and friendship ties were linked with nondisclosures. The most common mitigating strategies postulated were sensitisation, capacity building on parenting and the creation of child protection committees.

**Conclusion:**

From this study, nondisclosure of abuse is common in CWD, and thus there is a need for urgent attention to curb the situation for safer and more child-friendly environments through sensitisation, parental support and putting in place strategic child protection committees.

**Contribution:**

This article is based on the experience of all authors with interest in the field of disability. This article contributes to the pull of knowledge by providing context specific reasons for non-disclosure of abuse as well as mitigation strategies.

## Introduction

Abuse of children is a global human right and public health issue, with significant negative health and social impacts on children’s development. Child abuse is the abuse and neglect that occurs to children under 18 years of age. It includes all types of physical and/or emotional ill treatment, sexual abuse, neglect, negligence and commercial or other exploitation, which results in actual or potential harm to the child’s health, survival, development or dignity in the context of a relationship of responsibility, trust or power (World Health Organization [WHO] 2020). Estimate show that about 5% – 50% of children worldwide suffer from child abuse (Meinck et al. 2016). Estimates of 65% for physical abuse and 55% for both noncontact and contact sexual abuse have been reported in some African countries (Meinck et al. 2016). Although statistics are not readily available on the prevalence of children with disabilities in Cameroon, judging from the WHO (2011) estimates that 15% of persons in a given population live with a disability, it can be estimated that there are 3.7 million persons with disabilities in Cameroon (15% of the total population of 24 678 234) (Worldometer 2017). A previous study conducted in the Northwest Region of Cameroon shows an overall population prevalence of disability of 10.5% (95% confidence interval [CI] 9.0–12.2), with children 0–17 years at 4.7% and youth and adults 18–49 years at 6.8% (International Centre for Evidence in Disability [ICED] 2014). The 2010 African Child Policy Forum report on violence against children with disabilities in some African countries, including Cameroon, Ethiopia, Senegal, Uganda and Zambia, documented a very high level of violence against children. This report estimates that in Cameroon, over 50% of these children had been hit, punched, kicked or beaten; 25% made to eat hot chilli, pepper or very bitter food or drink; and over 25% choked, burnt or stabbed (Simo, Duthé & Odimegwu 2019). The situation is not different in other low- and middle-income (LMICs) countries, as the report goes further to state a relatively similar percentage of occurrences of abuse in children with disabilities (The African Child Policy Forum 2010).

Previous studies indicate high prevalence of child abuse in Africa and Cameroon (Danquah et al. 2015; Hildyard & Wolfe 2022):

[*W*]hile all children are at risk of being victims of violence, children with disability find themselves at a significantly increased risk because of stigma, negative traditional beliefs and ignorance associated with disability. (Hildyard & Wolfe 2002)

Anecdotal evidence suggests that local customs and beliefs exacerbate abuse, especially caused by insufficient awareness and action. The concept of child protection is relatively new in the Northwest Region of Cameroon, as most parents believe that they are supposed to bring up their children the way they want. Corporal punishment and other abuse are still rampant in schools and in the communities across the Region (Wango 2014). Evidence is essential to convince all actors of the need for change. All persons under the age of 18 have the same right as adults to physical and psychological integrity and to protection from all forms of violence (WHO 2020).

Article 19, page 3 of the Convention on the Rights of the Child (CRC) – adopted by the United Nations (UN) in 1989 – exhorts states to take:

[*A*]ll appropriate legislative, administrative, social and educational measures to protect the child from all forms of physical or mental violence, injury or abuse, neglect or negligent treatment, maltreatment or exploitation, including sexual abuse … (United Nations 2006a: Article 19, p. 3)

Similarly, the United Nations International Committee on the Rights of the Child has emphasised the importance of member countries prohibiting all forms of physical punishment and degrading treatment of children (CRC 2006). Nonetheless, for various social and cultural reasons, children and youth suffer abuse in the home (including foster homes), at school, in legal and child protection systems, at work and in the community (The African Child Policy Forum 2010).

Thus, children and youth are abused precisely in those spaces and places that should offer them protection, affection, developmental stimulation, shelter and promotion of their rights. One of the factors that make them highly vulnerable is their lack of autonomy, because of their young age and the consequent high levels of emotional, economic and social dependency on adults or institutions, which make it difficult for them to put a stop to the abuse, request help or report the situation (CRC 2006).

The Convention on the Rights of Persons with Disabilities (CRPD) is intended as a human rights instrument with an explicit, social development dimension (UN 2006). It adopts a broad categorisation of persons with disabilities and reaffirms that all persons with all types of disabilities must enjoy all human rights and fundamental freedoms. It clarifies and qualifies how all categories of rights apply to persons with disabilities, and it identifies areas where adaptations have to be made for persons with disabilities to effectively exercise their rights and areas where their rights have been violated and where protection of rights must be reinforced (UN 2006). The African Charter on Human and Peoples’ Rights (ACHPR) was adopted in June 1981 and entered into force in October 1986 to promote and protect human and peoples’ rights and freedoms while taking into consideration the legal and political cultures of African states, as well as preserving African tradition and identity (ACHPR 1986).

The government of Cameroon has been making great efforts in the past 25 years to protect the rights of children. This is evident in the signing of the CRC on 27 September 1990 and its ratification on 11 January 1993 (CRC 2000). The Cameroon government also endorsed the International Pact on Civil and Political Rights since 27 June 1984 and the Convention against torture and other cruel, inhuman or degrading treatments or punishments since 19 December 1986 (CRC 2000). Furthermore, Cameroon ratified certain legal instruments of a regional nature such as the African Charter on Human and People’s Rights on 20 June 1989 and the African Charter on The Rights and Well-being of the Child on 05 December 1997. Cameroon has also issued numerous laws, orders, decrees and circulars that are in accordance with the UN Convention (Lovert 2015). More so, Cameroon is in the process of completing a single, comprehensive national law specifically focused on children’s rights and welfare issues: the Child Protection Code (Lovert 2015).

Nationally, there exists a surfeit of laws for the protection of the rights of persons with disability (PWD). These laws include, *inter alia,* the much-heralded 1996 Cameroonian Constitution (The Government of Cameroon 1996) and the 2010 Law Relating to the Protection and Welfare of Persons with Disabilities (The Government of Cameroon 2010). However, these laws seem to remain only on paper with little or no practical implementation.

Child abuse, especially among children with disabilities, has been a common practice in Cameroon and the Northwest Region in particular for a long time (Wango 2014). Children, especially those with disabilities, were and are still considered people without rights, and their parents or guardians (including authorities under whose care they are) were supposed to bring them up in the way these parents or guardians desire (Mactaggart et al. 2016). These children encounter abuse of varied forms on daily basis, ranging from physical abuse to emotional abuse to mental and sexual abuse. When these children and young adults are abused, they may not disclose, some for unknown reasons, while some may just feel that when they complain no one will listen to them (Pinheiro 2006). Context-appropriate mechanisms have been advanced through research to ensure the respect of the rights of children with disabilities as enshrined in the United Nations CRPD (UN 2016), and the Child Protection Code under elaboration in Cameroon which will be a comprehensive national law specifically focused on children’s rights and welfare issues (United Nations 2006).

Child abuse, especially among children with disabilities, in Cameroon is rampant and a common practice and constitutes the violation of the most basic rights of children and youth as enshrined in the Universal Declaration of Human Rights. Surveys conducted across Africa and in Cameroon in particular show a high level of abuse against children and young adults with disabilities (CWD). For example, 52% of participants in Cameroon and Zambia reported being forced to have intercourse. These alarming rates do not include unreported cases of child abuse. The consequences associated with this do not only affect the victims but also the community as an entity. The prevalence of child sexual abuse in Africa ranges between 2.1% and 68.7% for girls in Tanzania and Ethiopia, respectively, and 4.1% – 60% for boys in South Africa (The African Child Policy Forum 2010). In some parts of Africa, more than 8 out of 10 children aged 1–14 years experience violent discipline every month. Africa has the highest rates of child neglect in the world, with 41.8% of girls and 39.1% of boys being neglected by their caregivers. In Nigeria, 66% of girls and 58% of boys under 18 witness violence in the home. More than half of all children aged 13–15 in West and Central Africa are bullied in school (The African Partnership to End Violence against Children *| End Violence* n.d.). This research investigates and documents in-depth information on the reasons and consequences of nondisclosure of abuse in children with disabilities and further develops appropriate and effective strategies to discourage such practices so as to sustainably improve the well-being of children with disabilities. Cameroon’s criminal justice system consists of two major departments, the bench and the criminal department, commonly known as the legal department, for prosecuting all criminal matters. The bench department consist of a residing magistrate for examining criminal cases, while the legal department supervises, controls and directs all investigations and prosecutes the same at different levels (Fonachu 2010). Investigation is regulated by the Criminal Procedure Code. Investigations are directly placed under the supervision of a magistrate acting as a state counsel. These investigations are carried out by the judicial police and gendarmes who act as auxiliaries of the state council (Fonachu 2010). The duties of the judicial police are performed by judicial police officers, judicial agents and all other legal and paralegal practitioners to whom judicial duties are assigned by law (Fonachu 2010). They are responsible for investigating offences, collecting evidence, identifying offenders and accomplices and bringing them before the legal department, including the abuse and violation of the rights of persons with disabilities.

There is a scarcity of data in Cameroon on the abuse, nondisclosure and mitigating factors of abuse in CWD. The aim of this study was to assess the reasons for nondisclosure of abuse and possible mitigating strategies among CWD. Thus, findings from this study will have a significant impact in the design and implementation of specific interventions on prevention and response programs to address abuse and violence against children with disabilities in the Northwest Region of Cameroon and even beyond.

## Research methods and design

### Study setting

The study was conducted in the Northwest Region of Cameroon, which is one of the country’s 10 Regions, with a population of about three million inhabitants (2015 extrapolation of 2005 National Demographic Census). The Northwest Region is divided into seven divisions and 32 subdivisions. According to the 2005 census, the region’s population is composed of 63% rural communities (Sneddon 2003). The study was conducted in five of the seven divisions in the Northwest Region, from which participants were selected. Two others could not be visited because of the prevailing crises which have led to so many people being displaced. People with disabilities and older people have been among those killed, violently assaulted or kidnapped by government forces and armed separatists, with several children undergoing one form of abuse or the other (Cameroon: People With Disabilities Caught in Crisis – Cameroon | ReliefWeb n.d.). The situation is likely to be worsened among children with disabilities.

### Study design

This study was a qualitative study involving the use of focus groups and in-depth interviews with CWD. In addition, key informant interviews were conducted among selected professionals in the domain of child abuse, family members, caregivers and other stakeholders.

### Study population

#### Children and young adults with disabilities who had experienced abuse

The Cameroon Baptist Convention Health Services (CBCHS) has a network of over 20 disability partner organisations, through which they provide financial assistance to enable CWD to attend school, receive medical intervention and find livelihood opportunities. The CBCHS is involved in strengthening child protection and safeguarding systems in the institutions and communities from which these children were enrolled in the study. Children and young adults (10–25 years) who had experienced one form of abuse or another were purposively selected for the in-depth interviews from these training and rehabilitation centres for persons with disabilities. These included six training and rehabilitation centres and five learning institutions. Study participants were also selected from communities in which these centres or institutions were located. Furthermore, children and young adults who had reported having experienced any form of abuse to any staff from the organisations or to a teacher, or a child who was observed to have experienced abuse, were purposively selected for the interview.

#### Parents of children or youth with disabilities who had experienced abuse

Parents of CWD were purposively selected from parent support groups affiliated with learning and training institutions for PWD where their children were enrolled. These parents resided in the communities.

#### Instructors or teachers of children and youth with disabilities

Instructors of CWD were purposively selected for the key informant interviews from six training and rehabilitation centres as well as from five learning institutions in which CWD were located.

#### Staff from relevant child protection offices

Community-based rehabilitation (CBR) staff, who visit the homes of children with disabilities on a daily basis to assess needs and provide appropriate interventions, were purposively selected for the interviews. These CBR workers from child protection offices signed child protection commitments to ensure they are not abusers, as they work with these CWD in the communities as well as within institutions.

#### Civil society and community members

Civil society members from partner organisations, who are involved in child abuse issues in the community either through counselling or provision of psychosocial support or linkages to legal and judiciary services, were purposively selected. Through civil society correspondence, community members were purposively selected based on their observation or experience of abuse of a child or young adult.

### Sampling strategy

#### Sampling technique sampling of children and young adults with disabilities

The Cameroon Baptist Convention Health Services Empowerment and Disability Inclusive Development (CBCHS-EDID) program works in collaboration with other organisations to support people with all types of disabilities from various institutions and communities. Purposive sampling was used whereby children and youth with disabilities from rehabilitation and training centres, in schools and in the communities who had experienced any form of abuse were recruited in the study. The research team also worked in collaboration with the CBR staff, who are in charge of identifying and assessing the needs of CWD in the communities and institutions, to identify those who had experienced or are experiencing any form of abuse. By assessing the needs of CWD over years, trust had been developed between these children and CBR workers, which made it easier for these children to confide instances of abuse to them. These children also disclose to their trusted friends. Thus, through these workers, the researchers were able to trace children and young adults who had experienced any form of abuse. Observations were also made during recruitment to determine if a child had experienced any abuse through body language or scars on the body. Safety was assured through working in collaboration with legal practitioners. The CBR program is also involved with the legal follow-up and safety of CWD experiencing abuse. Thus, both disclosed and undisclosed cases of abuse were followed up for reporting to guarantee the safety of the victims. Children and young adults were purposively selected to include all forms of disabilities (hearing, visual, physical and intellectual impairments).

#### Sampling of key informants

Key staff from child protection offices were also purposely selected to take part in the study. Information on reported cases of abuse or violence was obtained from Child Protection offices, Social Centres, the Justice and Peace office and school establishments where these children are enrolled in the study. Participants were purposively selected to represent all forms of disabilities in the study. As such, stratification was carried out to ensure that key informants in all the categories of disabilities were involved (hearing, visual, physical and intellectual impairments). Furthermore, family members, caregivers and community members were these children reside were also purposively selected into the study. Key staff from child protection offices were also purposely selected to take part in the study. Natasi (2014) recommends that a sample size for a qualitative study should be large enough to leave you with nothing left to learn, but not so large as to give room for redundancy (no new concepts emerging). With this in mind, the study’s sample size was therefore as follows: 50 children and youth with disabilities who had experienced abuse were recruited for the in-depth interviews. In-depth interviews were also conducted among 12 key informants. Furthermore, eight FGDs were conducted among key staff, parents and persons with disabilities from institutions and communities hosting these CWD, with an average of 6–8 participants per FGD. Data from interviews were collected based on the research questions until the saturation point was reached, that is, no new information could be obtained from the study participants.

### Data collection and study duration

Focus groups and in-depth interview guides were developed by authors J.C. and J.N., pretested and used for data collection based on questions on past studies and WHO standards on the types of abuse. Focus group and in-depth interview guides included, for example, the following questions:

- What are the various types of abuses you know?
- What are your general views about the abuse of children and young adults with disabilities in this community?
- Do you have examples?
- What can be done to reduce the abuse cases in children with disabilities?

Examples of questions for the in-depth interviews among CWD included:

- How do you feel as a child or youth when you are abused?
- What are the things people do to you that you do not like?
- Why did you hesitate to report the abuse?
- What can be done to prevent people like you from being abused?

Data were collected sequentially, starting with in-depth interviews among CWD who had experienced any form of abuse. Findings from these interviews were used to further refine the type of questions to include in the key informant in-depth interview and focus group discussion guides. Next, focus group discussions (FGDs) were conducted among staff from schools and relevant child protection offices, parents and persons with disabilities. Key informants with an in-depth knowledge on the topic under study were invited after focus group discussion sessions to take part in the in-depth interviews. Sequential data collection was done to refine the questions at each stage for the different groups of study participants.

Trained research assistants included sign language interpreters who assisted in the interviews of children and young adults with hearing impairments. Focus group and in-depth interview guides were pretested by trained data collectors on a separate set of children and young adults with different forms of disabilities, parents and key informants who were not part of the main study. Findings and responses from the pretest were used to modify the final data collection tools.

The data collection phase included in-depth interviews (among CWD both at home and in institutions) and Focus group discussions with parents of children with disabilities and key informants.

During interviews, observations were made to capture any facial expressions or behaviours that may further inform the findings of the study. Participants for in-depth interviews were recruited from six training and rehabilitation centres as well as from five learning institutions in which CWD were located.

Various techniques of interviewing children were employed. We used Keith’s (2013) recommendations of a comfortable setting, free from distractions, use of other play techniques to enable them to narrate or recall descriptions of how the abuse occurred without victimising themselves again and open-ended questions to get the best out of the child’s recall memory. The study was conducted over a period of three months from March to May 2018.

### In depth interviews among children and young adults

Interviews were conducted by J.N. with the assistance of trained data collectors recruited from the communities and institutions, including two sign language interpreters for sign language users, who had a one-on-one interview with all the 50 participants using an interview guide. This was done after the participants had received and signed an informed consent and/or assent form. Trained data collectors conducted interviews in collaboration with CBR staff (in charge of care and follow-up on legal procedures) and other partner organisation staff (Community of Practice for Gender and Child Protection, clinical psychologist, Community Counselling Clinic [CCC]) who work directly with these CWD. Thus, frequent visits to the homes and host institutions helped in building rapport and trust for smooth data collection. The interviews were conducted in the most rigorous way to ensure reliability and validity (‘trustworthiness’), thus ensuring credibility, transferability, confirmability and authenticity of research findings. This was done using more than one interviewer across the 50 interviews and corroborating narratives with observations. Transcribed data were read several times, and cross-validation of emerging themes was carried out among researchers. The research team is thus confident that the findings reflect the questions the research sought to answer. Fifty CWD were interviewed, including 25 girls and 25 boys. Only eight children with intellectual impairments were interviewed because of the difficulty in getting verifiable information from them, as shown in Table 1. For children below 18, an assent form was used. Authorisation to take part in the study was signed by caregivers of children. The study had no case of child abuse with the abuser being the parent. Thus, it was possible for a caregiver to give consent. For participants 18 years and above, consent was obtained from them directly. Both children, youth and adults were free to withdraw from the study when they wanted to, with no consequences for the care and treatment from the organisation. Various child and adolescent-friendly techniques were used to get more information from the children and adolescents.

**TABLE 1:** Characteristics of children and young adults with disabilities interviewed.

Disability	Age				Total	Male	Female
	10–13	14–17	18–21	22–25			
Physical	4	4	4	2	14	7	7
Hearing and speech	3	5	3	3	14	7	7
Visual	3	4	5	2	14	7	7
Intellectual	1	2	2	1	8	4	4

**Total**	**11**	**15**	**14**	**8**	**50**	**25**	**25**

### Key informant interviews

The participants were selected from both urban and semi-urban areas, and this permitted the research team to capture the type of abuse common among children and young adults in those areas. Interview guides were used to obtain information from key informants starting with general questions to more specific ones. In-depth interviews among key informants were conducted after completing those with CWD.

The key informants enrolled in the study included: parents of children with disabilities, CBR workers, persons with disabilities, legal practitioners, civil society members and community members.

### Focus group discussions

Eight focus group discussions, each comprising six to eight participants, were conducted among study participants. Participants were purposely selected and formal discussions were conducted using a guide and at a prearranged time and venue. Focus group discussions were conducted by J.N. as the facilitator with the assistance of trained data collectors, including a note taker. Each FGD lasted for 70–90 min. Information was obtained on the social structure of the community in which these CWD live, an in-depth understanding of the context and social fabric of the community and of how opinions and knowledge are formed in social contexts on child abuse. The FGDs were facilitated by a moderator (J.N.), who posed the questions using the topic guides and who was assisted by a note taker. The discussions were audio-recorded and transcribed immediately after the process. Observed body language and attitudes were also noted. Focus group discussion participants included female and male teachers of CWD, staff from relevant child protection offices, parents of CWD and persons with disabilities.

In the process of data collection, observations were carefully made to ensure that responses were consistent. For example, when a child said he or she had never been physically abused and scars were seen on his or her body, the research team probed further to know the origin of the scars. Also, during the focus group discussions, the team observed the process to make sure there was active participation from all to ensure that some participants were not dominating the discussions.

### Data analysis

The recorded data were transcribed verbatim and captured on an Excel database (Microsoft Corporation, Redmond, Washington, United States). Codes were given to similar themes guided by the key research questions on the reasons for nondisclosure and mitigation of child abuse among CWD. G.T.T., J.C.A. and J.N. undertook an analysis of the content of the open-ended responses using thematic analysis, which involves organising data into themes by recognising patterns (Braun & Clarke 2012). Data on the types of abuse were analysed by deductive coding, using a framework developed by the WHO on the types of abuse (WHO 2020) and inductive coding of emerging themes on the possible reasons for nondisclosures and proposed mitigation strategies. All qualitative data were read by C.W.A., who reviewed the analysis for consistency and quality. Themes and subthemes were refined through discussion between the researchers for group validation.

### Ethical considerations

Prior to the study, administrative authorisation was obtained from competent authorities in charge of the study institutions and communities visited and ethical authorisation was obtained from the institutional review board of the CBC health services. Apart from this, the study was conducted in line with the ethical principles for the conduct of studies with human subjects as stipulated by Beauchamp and Childress (1994), including autonomy, justice, nonmaleficence and respect of privacy and confidentiality of participants. The working context was carefully considered, knowing that it was a sensitive topic that could be traumatising to participants. As such, the services of a psychologist were sought. The trained research assistants conducted interviews, after which the CCC providing psychosocial counselling attended to each child or adult with disabilities. The CCC is headed by a psychologist and a certified clinical counsellor who were all enrolled during the data collection training stage of the study to prevent possible secondary trauma. The CBR staff in charge of following up with the legal course of abuse cases followed up with children and young adults who reported having had an experience or currently experiencing any form of abuse to prevent revictimisation.

All participants consented or assented to participate in the study and were free to withdraw at any time with no repercussions on them or on the services they were being offered. The research team had responsibilities to protect the research participants by involving legal practitioners and providing counselling for children who had experienced abuse. If a child had not disclosed any previous or ongoing abuse, the researchers ensured the safety of the child through maintaining contact with the child in collaboration with psychosocial counsellors, CBR workers and legal practitioners for justice to be done and to ensure the safety of the child or young adult with disabilities.

## Results

### Demographic characteristics of study participants

We had a total number of fifty (50) children and young adults with ages from 10–25 years. A total of 25 males and 25 females for the in-depth interviews with types of disability ranging from physical, hearing and/or speech, visual and intellectual impairments (Table 1).

A total of 12 key informants constituting six males and six females were interviewed from different institutions and in the community (Table 2).

**TABLE 2:** Characteristics of key informants interviewed in the in-depth interviews.

Category	Conducted	Male	Female
Parents of children with disabilities	2	1	1
Community-based rehabilitation workers	2	1	1
Persons with disabilities (experience and observed)	2	1	1
Legal practitioners	2	1	1
Civil society members	2	1	1
Community members	2	1	1

**Total number conducted**	**12**	**6**	**6**

A total of 60 participants took part in the focus group discussions from different institutions and in the community which constituted 29 males and 31 females (Table 3).

**TABLE 3:** Characteristics of participants in the group discussions.

Category	Number of group discussions conducted	Male	Female
1. Female instructors or teachers of children and young adults with disabilities.	1	0	8
2. Male instructors or teachers of children and young adults with disabilities	1	6	0
3. Female staff from relevant child protection offices	1	0	8
4. Male staff from relevant child protection offices	1	9	0
5. Female parents of children and young adults with disabilities.	1	0	8
6. Male parents of children and youth with disabilities.	1	8	0
7. Female group of persons with disabilities	1	0	7
8. Male group of persons with disabilities	-	6	0

**Total**	**-**	**29**	**31**

### Types of abuse experienced by children and young adults with disability

Results from the thematic analysis shows that the main types of abuse with various forms under each type were Physical abuse, Emotional abuse, Sexual abuse and Neglect (Table 4).

**TABLE 4:** Types of abuse experienced by children and young adults with disabilities.

Physical	Emotional	Sexual	Neglect
Corporal punishment or beating	Not going to school	Sexual intercourse	Food deprivation
Excessive labour	Abandonment	Touching child inappropriately	Lack of medical care
Stoning	Rejection	Exposure to adult pornography	Poor child care
Pushing	Name-calling	Exposure to other private parts	Neglect in education
Rubbing of pepper on child’s body	-	-	-

### Reasons for not reporting cases of abuse in children with disabilities

Thematic analysis from the group discussions and in-depth interviews from children and young adults with disabilities, parents and key informants shows several themes emerging as to the reason for nondisclosure of abuse, as presented below. In this presentation of findings, those from children and young adults with disabilities, parents and key informants are presented together.

#### Lack of awareness of where to report

According to the findings of this study, cases of abuse in CWD are rarely reported by the victims. Most of the participants said they were not aware of competent authorities to report to. This is partly because the few who have attempted to report to the police have most often not had fair responses. The participants suggested that a competent authority should be an institution that will take adequate actions against the abusers. An example of such quotes is presented below:

‘At times we want to report these abuses, but when we ask people where to report, they say they do not know.’ (IDI 17: Youth with disability, female, 19 years)

#### Long and expensive procedure

Most of the parents, key informants and young adults stated that the process to administer justice is long and expensive. They made mention of instances where they have reported cases to the police and other authorities but said authorities kept asking them to leave and come back. At times, they paid for transport to access these services, having to eat and forgo their business activities just to be asked to come back again, and even when they persevered with follow-ups, no favourable judgment was passed, and this discouraged them:

‘[… *S*]ometimes the process is complicated and takes a very long time. You will go and the police will tell you to come after and this goes on and on …’ (FGD: Parent 3, female, 43 years)

They also complained about the cost of hiring lawyers to defend their children in the courts. It also emerged that the participants do not know the procedure to report some cases of abuse, especially alleged rape. For the few cases of alleged rape reported, the participants said their cases were most often thrown out because they did not have medical certificates to prove that the child was raped. One of the parents state the following:

‘[… *T*]he cost for follow-up is very high; they will tell you that you should do medical certificate; you have to certify documents, pay transport up and down. It’s really discouraging …’ (FGD: Parent 2, male)

#### Lack of confidence in the justice system

The majority of the participants from the institutions as well as parents of CWD said it is needless reporting cases of abuse because the judicial and para-legal systems are corrupt. They recounted instances where people have reported cases of abuse and the abusers used their influence and money to corrupt the officials. A respondent said:

‘It is no secret that our judicial system is corrupt …. We reported a case of alleged rape with a medical certificate attached, but to our surprise, the abuser was set free the following day.’ (IDI 2: CBR worker, male 38 years)

A male parent of a child with disability gave an example of how a prominent man in the community abused his child and the case was reported, but it was dismissed on the grounds that there was no proof. To him, the abuser had corrupted the police chief, as seen from his comments below:

‘I was surprised the police chief called me and said, “There are not enough proofs that this man abused your child.”’ (IDI 1: Parent of child with disability, male)

Reporting is also hindered by the wrong assertion that nothing shall be done to the abuser even if the case is reported. Because of the lack of confidence in the judicial system, some of the participants reported that victims of abuse at times wrongfully believe that they can never get justice from the competent authorities, so it is needless to report this abuse:

‘Even if they report, what will be done? We all know how corrupt our system is.’ (IDI 1: Community member, female, 35 years)

#### Inaction by the teachers and parents

Many CWD acknowledged that they were not willing to report when they are abused because no action will be taken by their instructors at schools and at the training institutions where they are located. Below is an example of a quote from a respondent:

‘When I am abused by other students and I report to my madam, they do not do anything to the students, so I stop reporting.’ (IDI 17: Youth with disability, female, 19 years)

She went on to say that the fact that nothing is done causes the children to abuse her more.

Furthermore, in communities people look down on CWD such that they ignore these children and youth with disabilities when they report cases of abuse. At times, when these children do report the abuse to adults, they instead further abuse the children:

‘I reported to one uncle that his children are abusing me that with my bended legs and he asked me if my legs were straight.’ (IDI 28: Child with disability, male, 14 years)

Another child said when his siblings are beating him and he reports to his parents, they do not punish the siblings. So to him, it is needless to report the abuse.

#### Shame and stigmatisation

Stigma was also identified as one of the reasons why abuse is not reported. Most of the participants said that abuse is not reported because the abused are ashamed to let people know what has happened to him or her, especially in the case of sexual abuse. To them, it is better to stay quiet and be at peace than to report and live in ridicule. According to participants, victims of sexual abuse are usually laughed at in the communities when people know that they have been raped, as in most communities of the region, rape is unthinkable and a curse to the family. A respondent who reported a case of sexual abuse had this testimony:

‘People in my village usually point at me when I am passing, saying, “That is the girl who was raped.”’ (IDI 26: Young adult with disability, female, 20 years)

According to participants, sex is still considered sacred and therefore should not be uncovered, especially when you are not married. Reporting a case of sexual abuse is indirectly telling the community members that you had sex. Consequently, these victims are ashamed to report it, and at times they want to protect their family name. Their peers stay away from them, insinuating that they are ‘spoiled’ and that they can influence them. Other parents and adults make it difficult for children who have been abused sexually by sending them away from their homes and warning them never to come close to their children:

‘Many children who are abused sexually feel shy to open up to us, and as such, many of these abuse cases go on unnoticed.’ (Legal practitioner 2, female, 40 years)

#### Threats from abusers

Threats from abusers were noted to have restrained the level of reporting of abuse. At times, these abusers threaten these children that if they report cases of abuse, they will abuse them more. Since these children are mostly left alone, they take these threats seriously. A child with a disability stated as follows:

‘The man! If I report, he will kill me and that I should remember that I am always alone in the house.’ (IDI 36: Youth with disability, female, 18 years)

Some of the abusers even promised to end their parents’ means of livelihood, like taking back their farmland if they reported the abusers, as seen below:

‘The farm we work belongs to the man who raped me. He told my mother that if she tells anybody about this abuse, he will take back his land from us. As a result, my mother begged me not to tell anyone about what happened.’ (IDI 20: Young adult with disability, female, 25 years)

Again, the abusers threaten these children that nobody will believe what they are saying because they have a disability. Most participants said this is true, especially for children with intellectual disabilities:

‘People in our communities hardly believe what a child with intellectual impairment is saying.’ (IDI 2: Civil society member, male, 44 years)

To them, the child does not understand what he or she is saying. So at times when these children report to their parents, their parents do not believe them.

#### Protection of family unity and friendship ties

The majority of staff from child protection offices and CBR workers stated that the abusers of these children are people who are close to them. When a child is raped by a family member, the family will prefer to solve the problem within the family in order that people will not know what transpired. They think that if the case is reported to an outside authority, it will disintegrate the family unity. As a result, some families overlook an incident of abuse in order to protect their family names, but in the process they are putting these children at risk of further abuse and harm. Most abuse cases within family circles were noted to have been concealed within the family, with no assurances of a change in behaviour from the abusers. Below are examples of such quotes:

‘[… *S*]ome of these children are being abused by very close relatives, and their parents keep it secret …’ (FGD: Participant 3, female, 32 years)

‘[… *W*]hen they are abused by a stranger, it’s different. But if by a close relative, most parents will prefer to resolve it within, keeping the law away.’ (IDI 2: CBR worker, female, 39 years old)

#### Acceptance of abuse state

Interestingly, some of the victims of abuse have accepted their situation and see it as normal for others to be abusing them. This is true with many children being called by derogatory names on a daily basis. They have come to accept the names and answer when others call them by these names. Below is an example of such quote:

‘Everybody calls me “Eboa” [*physically disabled person*], so to me, it does not bother me anymore.’ (IDI 16: Child with disability, female, 12 years)

Another area of acceptance was sexual abuse. Some female children and youth with disabilities, especially those with multiple disabilities, experience sex primarily through abuse and may not experience sex outside of the abuse. The cultural beliefs by some people that persons with disabilities (especially girls and women) are asexual scare people away from them. Some parents expressed happiness that their children with disabilities were raped, as they are now grandparents, since few people will legally get married to their children with disabilities. Furthermore, some young adult women with disabilities, in the quest to have children of their own, did not report sexual abuse. A respondent who had been a victim of sexual abuse acknowledged this, as seen below:

‘I cannot report him because if I do, he will not come back again … in this condition, just few people will want to get married to you … I will like to have my own children too, so I did not report him …’ (IDI 49: Young adult with disability, female, 21 years)

### Mitigating approaches of child abuse

From the study’s research findings, mitigation approaches were proposed by children with disabilities, parents and key informants in child protection centres.

#### Multifaceted approach to sensitisation

From the thematic analysis, the majority of key informants, parents and young adults with disabilities proposed that community sensitisation involving several approaches should be used to create awareness. According to participants, a one-time sensitisation campaign will not yield the necessary impact because attitude change is a gradual process. The following quotes highlight this:

‘Parents and community members should be advised to stop abusing us and beating us.’ (IDI 05: Child with disability, male, 12 years)

‘We are begging that you should continue talking to everybody in the community … because they are still beatings us in spite of the fact that people have been talking to them not to beat us.’ (IDI 24: Child with disability, female, 11 years)

A female participant with a physical disability proposed the inclusion of children and adults in abuse awareness campaigns as role models, as seen below:

‘Adults and children with disabilities should be included in all sensitisation campaigns. Why is it that only people without disabilities talk on our behalf?’ (FGD: Participant with disability 6, female, 30 years)

According to participants, if PWD told their stories, focusing on the support they received at home, school and in the community and how they overcame challenges from their peers and other people, this would go a long way to foster disclosure of abuse among CWD.

Many teachers of CWD proposed ‘open days’ as a means of sensitisation in the communities, where children with disabilities can exhibit their products and showcase their talents which could alter negative attitudes and abuse of persons with disabilities. In addition, they believe that persons with disabilities should be encouraged to also participate in mainstream open days as this will also help to build their self-esteem.

Below is an excerpt from one of the female teacher’s narratives in a group discussion:

‘These children with disabilities have hidden talents that need to be exposed through exhibitions.’ (FGD: Participant 7, female, teacher, 41 years)

#### Capacity building on parenting

From the study, it was also noticed that ignorance on how to deal with children with disabilities by parents resulted in neglect and other forms of abuse. This suggests a lack of support provided to parents that could be remedied through parent support groups whereby these parents share their experiences, identify abuse in their children, understand sign language for proper communication with children and also understand how to take legal action if their child or young adult is abused. Below are examples of such quotes:

‘Parents of children with disabilities should form groups whereby their skills can be developed.’ (IDI 1: Parent of child with disability, female)

‘[… *N*]ot every parent understands the needs of a child with an impairment. They need to actively take part and learn in their support groups.’ (IDI: CBR worker, female)

#### Community child protection committees

The majority of the parents and CBR workers proposed putting in place community child protection committees as a mitigation strategy. This was further corroborated by many CWD, as they think that reporting abuse is challenging given that there are no trustworthy avenues in the communities to report to when they are abused. Below are examples of such quotes:

‘These children do not know where to report cases of abuse, and at times when they report, no action is taken.’ (FGD 2: Male parent of child with disability, 45 years)

A child with hearing and speech impairment affirmed this by saying, ‘When I was raped and I reported to one uncle, he instead shouted at me and told other people, who started laughing at me’ (IDI 27: Youth with disability, female, 16 years).

#### Schoolchild safeguarding guidelines

Many CWD expressed a lack of awareness of the poor treatment they receive as being abusive and something which is punishable by law. To overcome such ignorance on the part of CWD, there is a need for awareness creation on signs of abuse and how to avoid putting them at risk of harm or abuse. Ignorance was expressed by one of the children with disabilities, as seen below:

‘At times when they are abusing us, we are not even aware, since we are alone most of the times.’ (IDI 11: Child with intellectual disability, male, 14 years)

Such training should address cases of abuse at home, in schools and in the community. More so, children accept abusive situations when they do not know their rights and therefore need to be trained on these rights. Awareness on abuse as an abnormal act perpetrated on them must also be created.

The need of for putting in place awareness guidelines on abuse for children in schools and institutions was corroborated by a majority of teachers and staff from child protection offices. According to them, all schools should develop child safeguarding guidelines under the supervision of competent authorities. This was reflected in the response as seen below:

‘[… *E*]ach institution is supposed to have child safeguarding policies or guidelines …’ (FGD: Participant 7, female teacher, 41 years)

Most adults and young adults with disabilities raised concerns on their passive participation in activities that should actively include them and thus create awareness and prevent them being abused. This was acknowledged by one of the participants as seen below:

‘[… *W*]e don’t know, as decisions are taken on our behalf without consulting us …’ (FGD: Adult with disability, female, 30 years)

## Discussion

From the review presented in the introduction, child abuse is still common in Africa and specifically in Cameroon. Despite the laws put in place by the UN, CRC, CRPD and the government of Cameroon, cases of child abuse still go undetected and under-reported. For example, a study among some college students with disabilities who were abused revealed that only 27% reported the incident (Findley, Plummer & McMahon 2016). This study assessed the reasons for nondisclosure of abuse among CWD as well as proposed mitigation strategies. Understanding these provides a useful framework for empowering CWD in order to expose any subsequent abuse and further develop context-specific mitigation strategies to curb abuse among persons living with disabilities. From this study, four types of abuse were identified among CWD using the WHO framework (WHO 2020). Types of abuse ranged from physical abuse (corporal punishment or beating, excessive labour, stoning, pushing, rubbing of pepper on child’s body), emotional abuse (not going to school, abandonment, rejection and name-calling), sexual abuse (sexual intercourse, touching child inappropriately, exposure to adult pornography, exposure to others’ private parts) and neglect (food deprivation, lack of medical care, poor child care, neglect in education). A systematic review in West Africa (covering Guinea, Niger, Sierra Leone and Togo), (Njelesani et al. 2018), a study by Jones et al. (2012) in the United Kingdom and a study in the United States of America (Wildeman et al. 2014) also reported similar types of abuse in children with disabilities.

### Reasons for nondisclosure

Lack of awareness on where to report cases of abuse was one of the major themes that emerged as to the reasons for nondisclosure. In the study area, there are social centres in all of the subdivisions that handle cases of abuse. However, most community members do not know about the existence of these services nor their location, and hence they do not know where to disclose to such social welfare institutions. Awareness raising on where to report such cases will promote disclosure among CWD.

Furthermore, despite a lack of awareness of where to report, when abuse is reported to family members, especially parents, they most often do not take the necessary actions against the abuser(s) to alter their behaviour. This is aimed at protecting the family status quo, and thus the abuse continues. Parental restrictive gatekeeping may hinder second or third parties from being involved in the reporting of abuse cases related to their CWD so as to prevent legal actions against a family member. Another study (Collings, Grace & Llewellyn 2016) has shown that parental gatekeeping sometimes hinders children with intellectual disabilities of their right to be heard on issues concerning their lives. Childhood experts suggests that the perspectives of children with intellectual disabilities should inform social policy and research (Collings et al. 2016). While it is encouraging that more children are consulted about matters of importance to them, some children’s voices remain silenced as a result of parental gatekeeping (Collings et al. 2016).

A further reason given by parents, key informants and some young adults for nondisclosure was the lengthy and expensive procedures. Most of these children who are abused come from poor families, and at times the abusers are influential and can afford to hire lawyers to defend them. Persons with disabilities may experience significant barriers to engaging with the criminal justice system, including reporting to the police and participating in investigations and court proceedings. As evident in the present study, such processes are lengthy and difficult, taking into consideration their impairment.

Some parents, teachers and CBR workers expressed a lack of confidence in the justice system, as reported cases of abuse are never taken seriously. According to participants, when they see abusers walking around the communities as free citizens, it discourages them from reporting subsequent cases. It was further revealed by the study that those from poor backgrounds are marginalised when it comes to dispensing justice in the region, and it is difficult for the abusers to be punished because most of the children with disabilities who are abused come from poor families. If the justice system is corrupt and leads to incorrect judgments against reported perpetrators of abuse, this might hamper subsequent disclosures.

Inaction by authorities within educational institutions was also given as a reason for the nondisclosure of abuse among CWD. Some institutions where such abuses occur may want to preserve the name of their institutions and thus do not take action against abuse cases that occur in the institutions. A similar finding was obtained in Zambia (Njelesani, Si & Swarm 2022), where abuse perpetrated predominantly by nondisabled school peers towards disabled children went unaddressed by schoolteachers and authorities. Beyond the institution level, children and young adults can be empowered to disclose cases of abuse among trusted social workers or CBR workers.

Stigma among children, especially young adults with disabilities, was another reason for nondisclosure. Taking into consideration that sex before marriage is considered a taboo in most communities and religious organisations in the region, victims are ashamed to report when they are sexually abused. They prefer to hide it, thereby encouraging the abuser to continue with the abuse. Past research (Banks et al. 2017) has shown that stigma is an important factor in such abuse, which hinders reporting to the appropriate authorities. There is still a lack of understanding of people with disabilities among the wider public, and oftentimes having a disability makes them objects of jokes or fear. Children with disabilities may, as a result, feel stigmatised and as such not report cases of bullying and other physical, sexual and emotional violence.

Threat from abusers was seen as another reason for nondisclosure. In most cases, those who abuse these children are more powerful economically and have the means to threaten them. Some of these CWD from poor homes felt threatened by close contacts who provide benefits to either their parents or themselves, making it very difficult for them to disclose. Inclusive training and capacity building of parents and children with disabilities are necessary so that such children and young adults undergoing any form of abuse from such abusers might be able to disclose it without fear of losing any benefits.

Acceptance and living with the abused was also seen as a barrier for nondisclosure of abuse in children and young adults. Sometimes they may be silent, since they may have little power and less credibility as reporters of crime; they often choose to remain silent by accepting to live with the condition. Beckie et al. (2011) showed that acceptance and choosing to be silent was a reason for nondisclosure. Furthermore, some men are ashamed to associate with girls or women with disabilities during the day and will instead choose to sexually abuse them when they are alone. The present study’s findings also show that some parents are ‘happy’ that their children with disabilities were raped, as they are now grandparents, since few people will legally get married to their children with disabilities.

In addition, as reported by some participants, some young adults with disabilities are happy that they were now parents (through sexual abuse), although single mothers. Capacity building and self-esteem should be prioritised among children and young adults to see themselves as part of society with the right to marry and also have children of their own.

Participants had not received any form of legal redress after having reported incidents of violence perpetrated against them, despite pledges in the Constitution to support people with disabilities.

Other studies show similar views as to the reasons for nondisclosure by children with disabilities. In a review, Lyon (1996) found that threats decrease the likelihood that children will self-disclose sexual abuse. Threats included physical harm to the victim and/or their loved ones (Kaufman, Hilliker & Daleiden 1996) or forecasting negative or dire outcomes for the victim, their loved ones and/or the perpetrator. Furthermore, Lyon (1996) reported that younger children were less likely to disclose abuse than older children. Children who are abused by a family member were less likely to disclose and more likely to delay disclosure than those abused by someone outside the family (Lawson & Chaffi 1992). Children who do disclose during forensic interviews compared to children who do not disclose in such contexts (yet concerns remain that they have been abused) were more likely to have parents (particularly mothers) who were more supportive (McElvaney 2013).

### Mitigating approaches

Participants proposed that a multidimensional strategy should be used to carry out continuous sensitisation on the rights of children with disabilities at home, in schools and in the wider community so as to reach a diverse audience. For this to be achieved, appropriate communication channels should be used, including: community radio, schools, churches, meeting houses, cultural events and parent support groups. Other avenues that have been used and should continue include national and international days reserved for disability issues. However, these sensitisations should go beyond being run once a year, as this is often not very effective, but should be done on regular bases. Furthermore, most awareness-raising campaigns in the Northwest Region have been led by persons without disabilities, although working in the disability community. This has its limitations and the study informants think that for such sensitisation to be successful, persons with disabilities should be used as role models during awareness-raising campaigns.

The research team found out from participants that a picture storybook on the study findings would be an effective sensitisation and education material and should therefore target a wide range of stakeholders. This has the potential of reaching a wider public than the sensitisation campaigns on international days, mostly attended by people who are already aware of disability issues. In effect, most books that children with disabilities access seldom have stories of adventures by children with disabilities nor do they even make reference to them. This storybook could be adapted in various versions to meet children’s, youth and adult expectations.

The formation of parent support groups and capacity building was also suggested as strategies to curb child abuse among CWD. Members of these parent support groups should also serve as peer educators to other parents. This will reinforce positive attitudes and provide a place to share experiences, which will help them overcome many of the challenges in parenting children with disabilities.

The parent support groups could further become avenues to enhance communication skills for children with disabilities. For instance, sign language could be taught to parents and other community members to ensure easy communication with children with hearing impairment and identification of signs of rape in their children with disabilities. Also, during the training of parent support groups, they should be sensitised on the need to assign responsibilities to children with disabilities that are appropriate to their age in order to prevent the incidence of child labour – also a form of abuse.

Putting in place child protection committees was also suggested by most CBR workers and parents as a mitigation strategy. The majority of children with disabilities who have been abused thought that if there is a reliable place where they can report abuse, they will feel comfortable reporting it. In this light, a child protection committee should be formed and trained in the communities, to whom children can report cases of abuse. Such structures will be inclusive, receiving complaints from all children, including those without disabilities, and transmitting them to appropriate structures as necessary.

Many teachers and staff from the relevant child protection institutions recommended putting in place child safeguarding policies on abuse to be taught in schools. As part of such guidelines, child safeguarding commitments should be signed by all staff and supported with relevant training on safeguarding principles.

Children in schools with guidelines should be regularly educated on their rights, safety tips and procedures for reporting abuse. As part of safeguarding guidelines, friendship groups comprising children with and without disabilities should be created in schools to foster peer-to-peer support, prevent bullying, reduce stereotypes and build the self-esteem of children with disabilities. This measure has the potential to dismantle attitudinal barriers.

According to some participants, their involvement in activities concerning them will help create awareness and prevent abuse in children with disabilities. Hence, measures should be taken to include children with disabilities in all mainstream activities by involving them in planning, implementation and monitoring of activities that involve them. Supporting education for children with disabilities is a key strategy. Strategies should be put in place to ensure children and adults with disabilities are taken seriously and also given opportunities to build their own self-esteem and empowerment which may allow them to resist being abused.

Banks et al. (2017) propose disability-inclusive planning to prevent abuse in children with disabilities. An important part of creating lasting change will be welcoming the participation of more people with disabilities, including young people, in policymaking. Many people lack knowledge and training about how to identify and respond to the unique dynamics and contexts that arise when there is abuse in CWD. Misunderstanding or ignoring these unique dynamics proves costly to the safety and healing of victims with disabilities.

Multidisciplinary working can provide a positive context for fairness where it is well coordinated, with effective communication and information sharing. Ofsted (2012) found that multi-agency support at an early stage is valuable in tackling emerging concerns about children with disabilities. Staff with expertise in child protection institutions may identify concerns overlooked by colleagues with disability expertise and vice versa (Ofsted 2012). Work with children with disabilities is not always well-coordinated, thus increasing the danger of abuse being under-reported.

Child welfare professionals, parents and teachers should be aware of protective factors associated with CWD. Protective factors are conditions or attributes in individuals, families or communities that can mitigate or eliminate nondisclosures that decrease the health and well-being of children and families. For example, an increased willingness on the part of parents and teachers to engage with various service professionals (a protective factor) could safeguard CWD who would otherwise be at risk of abuse (Haight et al. 2013). Studies show that a focus on strengths can help improve children’s self-esteem and increase disclosure of abuse. When child welfare professionals work with families of children and young adults who have disabilities, this type of strengths-based approach allows the child to feel supported and can reduce the risk of abuse as well as exposure of such abuse (Lightfoot 2014). Building strong, positive relationships with families and focusing on caregivers’ strengths can also improve parents’ confidence and self-esteem, which can reduce stress, risk factors of abuse and empower them to prevent or report any future abuse (Algood, Harris & Hong 2013).

## Conclusion

This study was aimed at identifying reasons for nondisclosure of abuse in children with disabilities as well as possible mitigating strategies in the Northwest Region of Cameroon. From these findings, reasons for nondisclosure of abuse range from lack of awareness on where to disclose, fear of poor treatment from the abusers and the acceptance of the abuse condition. Several mitigating approaches were postulated from the participants which, if implemented, will avert or curb abuse of children with disabilities. These approaches advocated for policies to curb these abuses, sensitisation, capacity building on parenting and the creation of child protection committees. Urgent attention is therefore needed to empower CWD so that they will be able to protect themselves from abuse and also report any abuse they experience. There is a need to reinforce policies to curb the situation and create a safer, child-friendly environment. Organisations for people with disabilities should condemn and report all instances of abuse against CWD and monitor the observance of all national and international laws governing such abuse. Persons with disabilities should be included in planning and decision-making platforms which concern them, as this will help create awareness on abuse and reporting of such abuses. Furthermore, there is a dire need for coordination mechanisms to ensure efficient and effective implementation of all proposed interventions.

### Recommendations for future studies

Limited studies are available from the literature, suggesting the need for more evidence-based research and the prioritisation of abuse of CWD by the national and global health community. Apart from various reasons for nondisclosure of abuse as obtained from this study, more studies are needed to assess context-specific risk factors of abuse among CWD.

### Study limitations

There is a possibility of under-reporting of the reasons for nondisclosure of abuse because of recall bias, since this study focused on past reports and suspected abuse among CWD. However, probing and follow-up questions were asked for them to recall past experiences.
